# Correction Notice: Selecting β-thalassemia Patients for Gene Therapy: A Decision-making Algorithm

**DOI:** 10.1097/HS9.0000000000000622

**Published:** 2021-07-20

Since the publication of the article entitled “Selecting β-thalassemia Patients for Gene Therapy: A Decision-making Algorithm” (*HemaSphere.* 2021;5(5):e555), the authors inform the readership that Achille Iolascon, based at the Dept. of Molecular Medicine and Medical Biotechnology, University of Naples Federico II, and at CEINGE Biotecnologie Avanzate, Naples, Italy, should be acknowledged as an author. This has been updated.

The changes have been made online: https://journals.lww.com/hemasphere/Fulltext/2021/05000/Selecting_thalassemia_Patients_for_Gene_Therapy_.12.aspx

